# Eradicating polio in Pakistan: an analysis of the challenges and solutions to this security and health issue

**DOI:** 10.1186/s12992-016-0195-3

**Published:** 2016-10-12

**Authors:** Shoaib Fahad Hussain, Peter Boyle, Preeti Patel, Richard Sullivan

**Affiliations:** 1 Conflict and Health Research Group, King’s Centre for Global Health, King’s College London, Suite 2.13 Weston Education Centre, Cutcombe Road, London, SE5 9RJ UK; 2 International Prevention Research Institute, Lyon, France; 3 University of Strathclyde Institute of Global Public Health @iPRI, Lyon, France; 4 Department of War Studies and Conflict and Health Research Group, King’s College London, London, UK

**Keywords:** Polio, Pakistan, Conflict and health, Vaccine coverage, Socioeconomic factors, Global health

## Abstract

Since the launch of the Global Polio Eradication Initiative (GPEI) in 1988 the global incidence of poliomyelitis has fallen by nearly 99 %. From a situation where wild type poliovirus was endemic in 125 countries across five continents, transmission is now limited to regions of just three countries – Pakistan, Afghanistan and Nigeria. A sharp increase in Pakistan’s poliomyelitis cases in 2014 prompted the International Health Regulations Emergency Committee to declare the situation a ‘public health emergency of international concern’. Global polio eradication hinges on Pakistan’s ability to address the religious, political and socioeconomic barriers to immunisation; including discrepancies in vaccine coverage, a poor health infrastructure, and conflict in polio-endemic regions of the country. This analysis provides an overview of the GPEI, focusing on the historical and contemporary challenges facing Pakistan’s polio eradication programme and the impact of conflict and insecurity, and sheds light on strategies to combat vaccine hesitancy, engage local communities and build on recent progress towards polio eradication in Pakistan.

## Background

Before the launch of the Global Polio Eradication Initiative (GPEI) in 1988, poliomyelitis paralysed and killed hundreds of thousands of people every year [1]. Polio is highly infectious, consisting of serotypes 1, 2, and 3. Horizontal transmission usually occurs via the faeco-oral and oral-oral routes, with faeco-oral transmission contributing to the majority of cases especially in regions with poor hygiene and sanitation [2, 3]. The development of the inactivated poliovirus vaccine (IPV) in 1955, led to a sharp reduction in viral transmission and cases in the USA from approximately 20,000 cases per year in the 1950s to less than 1000 cases by the 1960s [4]. However, the live-attenuated oral poliovirus vaccine (OPV) developed in 1963 remains the major vaccine in the developing world due to its cost-effectiveness, easy oral administration and comparatively better induction of intestinal immunity compared to the IPV [5].

The use of these vaccines rapidly interrupted the indigenous transmission of wild poliovirus in the Americas by the early 1980s and led to the launch of the GPEI with a resolution of the World Health Assembly (WHA) in 1988 [6]. The GPEI has made extraordinary progress since 1988; from a situation where wild poliovirus was endemic in 125 countries across five continents, paralysing 350,000 children every year, to reducing global annual incidence by more than 99 % [1]. In addition, wild type 2 poliovirus was successfully eradicated in 1999 and the last case of wild type 3 poliovirus was reported in November 2012 [5, 7]. In 2015, four of the six WHO regions were certified as polio-free, leaving Pakistan and Afghanistan as the remaining endemic reservoirs of wild poliovirus [8]. Illiteracy, religious misconceptions, conflict and insecurity in these regions have seriously hampered vaccination efforts and have been directly associated with a rise in wild poliovirus transmission with the potential to unravel the progress made by the GPEI [9]. These fears were realised following an outbreak of 36 cases of polio in Syria and the isolation of wild poliovirus type 1, traced to Pakistan, from sewage samples in Israel towards the end of 2013 [10, 11]. The successful eradication of wild poliovirus therefore rests on Pakistan’s immunisation programme and strategy.

This review focuses on Pakistan’s polio eradication campaign in the context of its current security environment. It aims to highlight (1) the historical challenges faced by the GPEI (2) obstacles faced by Pakistan in its polio eradication campaign and (3) lessons and solutions learned from the campaign in a conflict health environment.

## The Global Polio Eradication Initiative

The GPEI is a multinational partnership between the World Health Organization (WHO), the United States Centers for Disease Control and Prevention (CDC), The United Nations Children’s Emergency Fund (UNICEF), Rotary International and national governments. Polio-affected countries undertake all eradication activities with major strategic planning, coordination and data collection carried out by the WHO [6].

Despite the launch of the GPEI in 1988, most endemic countries only began polio eradication programmes in the mid-1990s, with the last two countries, the Democratic Republic of Congo and Sierra Leone only starting national polio eradication campaigns in 2000 due to civil conflict, the initial target for global polio eradication [6]. The GPEI has therefore had to operate in a complex and ever-changing geopolitical landscape and contend with many factors particularly the impact of conflict.

However, it would be wrong to assume that conflict alone has been the main barrier. In many cases, polio-endemic countries have faced numerous additional challenges culminating in insufficient population immunity to halt viral transmission. The implementation of Supplementary Immunisation Activities (SIAs) has been a cornerstone of the GPEI’s strategy to sufficiently raise population immunity and halt transmission. This strategy has been difficult to implement in some regions due to geographical limitations, such as in the Kosi river basin in Bihar, India. Poor management and health communication strategies have also been a hindrance, as documented in Northern Nigeria and Pakistan, where provincial and district health agencies, which controlled most vaccination resources, were not sufficiently engaged nor were religious and community leaders advocated to support the campaign [6]. Misinformation and suspicion regarding the OPV campaign in some communities have also been a significant barrier to achieving the GPEI’s goals. These concerns include misconceptions regarding the efficacy of the OPV in local communities and among vaccinators engaged in repeat SIAs. A widespread belief documented in Nigeria, India, Pakistan and elsewhere that the OPV causes infertility as well as cultural barriers such as the deployment of all-male vaccinator teams in conservative regions have also been highlighted [12].

As the global incidence of wild poliovirus falls, the widespread use of the live-attenuated OPV in developing countries has been associated with reversion of live-attenuated virus to virulent form, a circulating vaccine-derived poliovirus (cVDPV) [13]. Traditionally, outbreaks of cVDPVs and vaccine-associated paralytic poliomyelitis (VAPPs) have been controlled with increased surveillance and SIAs using OPVs, with IPV use restricted in regions prone to faecal-oral spread. The risk of accidentally releasing wild poliovirus from IPV production facilities, as was the case in The Netherlands (1992) and Belgium (2014), has resulted in limiting production to a few industrialised countries where the consequences of releasing poliovirus into a crowded region with poor sanitation is diminished [14]. Insecure, conflict-affected countries have a particular risk profile in this regard. However, the current prevalence of VAPP and cVDPVs requires the phased withdrawal of all OPVs if all sources of poliomyelitis are to be eliminated. This is reflected in the 2013–2018 Polio Eradication and Endgame Strategic Plan, which calls for the synchronised replacement of tri- and bivalent OPV by April 2016 upon the introduction of IPV in routine immunisation across countries [15, 16].

## Impact of conflict on the GPEI in Pakistan

### Pakistan’s polio eradication campaign

Pakistan’s polio eradication programme has come under international scrutiny due to its position as the main driver of global wild poliovirus spread in recent years. This is rooted in financial and organisational deficits, as well as active conflict and insecurity, which has resulted in the persistent failure of effective immunisation campaigns and SIAs to reach all areas of the country.

Pakistan is divided into four provinces (Sindh, Punjab, Khyber Pakhtunkhwa [KP] and Balochistan), the Islamabad Capital Area, the Federally Administered Tribal Areas (FATA) and two administrative areas (Gilgit-Baltistan and Azad Jammu and Kashmir) [17]. It is the most populous country in the WHO’s Eastern Mediterranean Region and one of the least developed, with a large population of approximately 188.9 million people including 24.7 million children under 5 years old and 66.2 million children under 15 years old, making it critical to polio eradication efforts [17, 18].

Pakistan’s Expanded Programme on Immunisations (EPI) was formally initiated in 1978 after the WHO launched its Expanded Programme on Immunisation in 1974 [19, 20]. National EPI policy is regulated by the guidance of the National Immunisation Technical Advisory Group (NITAG), which makes evidence-based recommendations to improve the programme [19]. The immunisation programme is delivered with the assistance of 10,000 vaccinators, 6000 Lady Health Visitors and other paramedical staff. In addition, over 100,000 Lady Health Workers support the programme through social mobilisation efforts, tracing vaccine defaulters and occasionally administering vaccines. There are approximately 6000 fixed EPI centres but their variable geographic distribution at district and sub-district levels make national immunisation drives and SIAs the mainstay of polio eradication efforts [21]. The polio eradication effort in Pakistan is also funded and supported by a number of international organisations including the WHO, UNICEF, the Bill and Melinda Gates Foundation and the Global Alliance for Vaccinations and Immunisations (GAVI) [19].

### Immunisation and surveillance strategies

Routine immunisation against poliovirus and five other diseases was initiated in 1978 with the launch of the national EPI. However, SIAs specifically targeting polio began in 1994 in line with the GPEI, with the strategy evolving over the past two decades to intensify campaigns during seasons when the risk of viral transmission was high, and SIAs in border regions were coordinated to coincide with SIAs in neighbouring Iran and Afghanistan for maximum coverage [22].

From 2000 onwards, the campaign strategy incorporated house-to-house visits with at least 7 rounds of national immunisation days and targeted ‘mop-up’ SIAs during negotiated ceasefires in conflict regions, based on local surveillance results or in response to disease outbreaks. OPV type has also changed over time to reflect changing WHO recommendations and innovations in vaccine composition with trivalent OPVs were used exclusively in SIAs from 1994 to 2004, the introduction of monovalent OPV types 1 and 3 in 2005 and 2007 led to their use in combination with trivalent OPVs in SIAs from 2005 to 2010. Monovalent OPVs were replaced with the introduction of the bivalent OPV in 2010 for subsequent campaigns [17].

National surveillance of acute flaccid paralysis (AFP) was also initiated in conjunction with vaccine delivery in 1994 although the necessary infrastructure and training programmes only became functional in 1998. In addition to adopting AFP diagnostic criteria in the field, the National Institutes of Health in Islamabad serves as the WHO-accredited national poliovirus laboratory where stool and sewage samples are analysed to monitor the efficacy and sensitivity of AFP surveillance and provide genetic data on circulating poliovirus lineages [23].

### Barriers towards polio eradication in Pakistan

There is a strong correlation between low immunisation completion and negative socioeconomic factors, in addition to conflict, such as illiteracy, poverty and difficulty accessing community health and immunisation services [19]. Pakistan faces all of these challenges coupled with a difficult geography, from the Himalayan mountain range and glaciers of the north to the harsh terrain of Balochistan in the south, contributing to poor public health delivery. Vast differences in the country’s population density also present challenges to the polio eradication campaign; with densely populated cities such as Lahore and Karachi presenting the risk of rapid faecal-oral spread juxtaposed with the sparsely populated and heavily mountainous Balochistan province [24].

### Poor management and operational deficits

With less than 2 % of the Gross National Product (GNP) spent on healthcare, an adequate health infrastructure and service delivery system is severely lacking in many parts of the country. Although the GPEI’s Pakistan operation is relatively well-financed, its efficiency is compromised by a lack of transparency in governance, an under-resourced public health delivery service and a poorly regulated private health sector [25].

Closser [26] notes that with more pressing national problems such as frequent power outages, the lack of running water in many areas, the loss of agricultural output and food supply from natural disasters and active insurgencies in Balochistan, KP and FATA; health initiatives such as implementing the GPEI have had a lower priority especially compared to the resource demands of combating more prevalent infectious diseases. This is compounded by poor management and allocation of finances at the regional/district level, leading to inefficiencies in vaccine delivery and a pervasive lack of accountability in meeting immunisation targets [19].

There are also significant inequities in immunisation coverage and resource availability at the provincial and district levels, with conflict and insecurity affecting access in some regions; immunisation coverage varied from more than 75 % in Punjab to less than 45 % in FATA and Balochistan [27]. This extends to large urban centres such as Karachi, where low-income neighbourhoods had significantly lower coverage compared to more affluent areas of the city [19]. Furthermore, the central government’s influence in regulating and implementing the polio eradication campaign is highly variable with reports of district health officials resisting directives from superiors through false compliance, refusing to work or direct confrontation, with one report suggesting that vaccinator teams had a 31 % higher chance of not visiting a target area than the baseline vaccine refusal rate [28]. Some EPI centres are also reported to lack vaccination registration records or computerised immunisation registries, and with the last census conducted 16 years ago, these present significant hurdles in estimating the number of children needing vaccination [19]. Vaccinator satisfaction has also been negatively affected by systemic flaws in the EPI including the lack of financial incentives, low salaries and delays in payment [29]. These factors have all led to an environment where institutionalised malpractice is commonplace, with inspectors appointed on political grounds rather than experience, and the active pilfering of resources from the state [25, 28].

### Concerns regarding OPV efficacy

OPV use correlates with the incidence of cVDPVs and VAPPs especially in regions prone to enteric infections such as Pakistan. Frequent power outages and the scarcity of equipment in Pakistan have made it difficult to maintain the cold chain necessary for OPV efficacy, and has contributed to the alarming rise of cVDPVs as well as wild poliovirus-induced poliomyelitis among vaccinated children [27, 30].

### Vaccine hesitancy

Pakistan’s basic adult literacy rate is approximately 60 % with higher rates in urban centres than rural areas, where two-thirds of the population live [19, 28]. Illiteracy, socioeconomic, cultural and religious factors have all contributed to vaccine hesitancy in Pakistan. Parental refusal is a significant hindrance to the vaccination campaign due to misconceptions regarding the purpose or effectiveness of immunisation such as the widespread misconception that vaccines can harm or sterilise children, or contain monkey- or pig-derived products which is forbidden in Islam. The repeated administration of the OPV was also cited as a barrier; with some parents suspecting that this was to ensure that their children were sterilised, or that substandard vaccines were being used [31].

Cultural issues such as the presence of all-male vaccinator teams when the mother is alone, or when family or community elders have not given consent for vaccination have also been cited as important barriers to immunisation in some communities [19]. A basic lack of awareness of the symptoms and effects of polio in target populations is also to blame for vaccine refusal, leading to damaging rumours including the misbelief that polio-associated paralysis is curable [9]. However, the persistence of poliomyelitis in KP and FATA, the major wild poliovirus reservoirs in Pakistan, is intimately linked to active conflict and insecurity in these regions.

### Conflict, militancy and the polio eradication campaign

Despite the multitude of challenges facing Pakistan’s polio eradication campaign, the annual number of polio cases has declined by more than 90 % since 1994. The strategy of combining routine immunisation and SIAs led to a significant reduction of cases between 1998 and 2005, however poliomyelitis cases began to increase from 2006 as the Taliban insurgency intensified in KP and FATA, leading to the displacement of millions of people and inward migration to major urban centres such as Karachi [17, 30].

Conflict and insecurity in KP and FATA led to a dramatic rise of reported paralytic polio cases in Pakistan, from 91 cases in 2013 to more than 300 in 2014, representing more than 85 % of the global wild poliovirus caseload. In response, the International Health Regulations Emergency Committee declared the situation a public health emergency of international concern in 2014, even considering an international travel ban to reduce the risk of wild poliovirus spreading to polio-free countries [14, 30, 32]. Recent outbreaks of poliomyelitis in central Asia, central Africa and the Middle East, which were traced to either Pakistan or Nigeria, underscores the importance of continuing the polio eradication effort in these regions [9, 14]. The following sections explore how conflict and militancy have affected Pakistan’s polio eradication campaign in greater depth.

### Negative propaganda against vaccination

A concerted propaganda campaign by militants operating from the Pakistan-Afghanistan border region, and supported by some religious clerics, has spun a narrative linking vaccination programmes to a Western plot to sterilise Muslims and painted vaccinators as spies for the US Central Intelligence Agency’s (CIA) highly unpopular drone programme; especially after revelations that the CIA funded a fake hepatitis B vaccination campaign in Abbottabad to trace Osama bin Laden [28, 30, 33]. This has led to a deep suspicion of the polio eradication campaign, especially in rural FATA and KP, which have the highest vaccine refusal rates and present with the most poliomyelitis cases [9, 17]. Vaccinators operating in conflict-affected parts of FATA and KP report significant hostility, partly because they are perceived to be following a Western agenda [31, 34]. Survey data from FATA showed that only 25 % of residents trusted vaccinators compared to 61 % in low-conflict areas, highlighting how pervasive this narrative has become [9].

There is some evidence that attitudes and perceptions towards polio vaccination are shifting, with recent cross-sectional studies suggesting that the majority of the public in a sample from Punjab support the immunisation programme, although some reticence does remain on religious grounds [35]. This contrasts with findings from a cross-sectional study of residents of Quetta and Peshawar, both highly affected regions, where false religious beliefs, fears of vaccine-induced infertility and security issues were identified as major barriers towards acceptance [36].

### Vaccination bans and security concerns

The Taliban-imposed ban on vaccination in 2012 has been detrimental to polio eradication efforts, especially in parts of FATA where more than 350,000 children remained unvaccinated for more than 2 years [17]. Targeted attacks against immunisation teams since 2012 have tragically killed scores of vaccinators, curtailing the implementation of SIAs and house-to-house visits and the cancellation of post-SIA surveys and quality assessment in high-risk areas [37, 38]. The prevailing strategy in these areas has had to shift due to security concerns; limited SIAs are now only carried out under armed escort in one day without giving residents prior notice [17]. This climate of fear and insecurity has also impacted the recruitment, training and retention of vaccinators; with FATA and KP-based teams now facing a significant shortage of trained staff [27].

Fighting between Pakistan’s military and insurgents operating from polio-endemic regions has resulted in the internal displacement of millions of people, and with it, the spread of wild poliovirus to other parts of Pakistan and other countries. AFP and environmental surveillance data indicate that current wild poliovirus transmission originates from these regions in FATA and KP, with a significantly higher prevalence among internally displaced persons (IDPs) settled elsewhere in the country [17, 27].

### Recent progress towards polio eradication

In 2015, territorial gains made by the Pakistani military in FATA, gave more access to vaccinator teams in regions where they were banned [28]. The KP provincial government also initiated the ‘Alliance for Health’ programme in February 2015 to improve security for vaccination teams and increase community engagement efforts in FATA [39]. Collaborations between Pakistan’s security forces and the polio eradication campaign have helped achieve a reduction of over 80 % in new polio cases reported in 2015 [40] compared to 2014 (306) [41, 42].

### Strategies to improve the polio eradication campaign

Pakistan’s National Emergency Action Plan for Polio Eradication (NEAP) 2015–2016 reported that deficits in the quality and coverage of the polio eradication campaign including suboptimal AFP surveillance, delayed payments to staff, poor accountability and management practices and the widespread disruption of SIAs in polio-endemic regions due to attacks on vaccination teams and their security escorts led to a large number of children, more than 80 % of them under 2 years old, remaining unvaccinated in 2014 [43].

The NEAP outlines steps taken by Pakistan’s government to improve polio vaccination outcomes including overhauling the polio eradication campaign’s management structure to increase accountability and transparency and improve vaccinator training, recruitment and retention. It also highlights efforts to address low vaccinator morale by offering more guidance and support to frontline staff, improving the current payment mechanism, reducing staff turnover rates and establishing minimum performance benchmarks at the Union Council level. Improving existing security protocols for vaccinator teams during and prior to immunisation campaigns in coordination with military and law enforcement agencies has also been identified as a key priority in high-risk areas [43].

### Strengthening Pakistan’s health infrastructure

Investing in Pakistan’s EPI programme is critical to achieving polio eradication in the country. In addition to misconceptions and concerns regarding vaccination, one of the main reasons for refusal has been the lack of access to EPI centres [19, 44, 45]. Increasing the number of fixed EPI centres, especially in rural areas, is crucial to effective service delivery in hard-to-reach regions of the country. Pakistan’s 100,000 female health workers and military personnel operating in high-risk areas can also be trained to administer vaccines that can help mitigate the current shortage of vaccinators [19, 46]. Accountability and institutionalised malpractice have also been cited as major issues facing the EPI programme, and steps must be taken to ensure that district health officials are adequately trained and evaluated to ensure sufficient vaccine coverage in a given district.

Owais and colleagues [19] also note that despite EPI centres being hosted in Basic Health Units (BHU), there is a lack of integration between vaccination and curative health services. Furthermore, there are also discrepancies in immunisation coverage for the more prominent polio eradication campaign and campaigns against other infectious diseases such as measles, diphtheria, pertussis and tetanus (DPT); in fact, children are more likely to be vaccinated against polio than DPT or measles [1]. Integration between these arms of Pakistan’s health services and their delivery as an inclusive package can be a more effective way to engage communities and religious leaders than a narrow, polio-specific agenda [46].

### Community engagement and education

The fallout from the CIA’s fake hepatitis B vaccination campaign in 2011, and the politicisation of vaccination campaigns has made the creation of ‘days of tranquillity’, ceasefires brokered in previous conflict zones for vaccine delivery, a distant possibility [46, 47]. Visible partnerships between political and religious authority figures have been instrumental in Nigeria's polio eradication campaign [48]. Ending the 2003 polio immunisation boycott in Northern Nigeria required intensive diplomacy; the United Nations (UN) sent a special envoy to negotiate ending the polio boycott with key religious and political leaders, and the GPEI worked with the Organisation of the Islamic Conference (OIC) to address concerns regarding the aims of the polio eradication campaign, as well as the safety and efficacy of the polio vaccine. Saudi Arabia’s enforcement of WHO recommendations with *fatwas* (formal Islamic rulings) to vaccinate pilgrims undertaking the Hajj in 2005 also helped dispel suspicions that the polio eradication campaign aims to sterilise Muslims [49]. This strategy has also been applied in Pakistan where prominent Islamic scholars issued a *fatwa* endorsing the polio vaccination campaign [50]; and have also led door-to-door campaigns in parts of the country [36].

The health communication strategy in India and Nigeria incorporated sustained media campaigns, the support of influential public and religious leaders and efforts to overcome social barriers to provide vaccine coverage to vulnerable and hard-to-reach populations, which has translated to a situation where these countries are now polio-free [5, 12]. Gaining support from influential public figures and the use of mass communication strategies for health awareness and social mobilisation can help shift negative perceptions of the polio vaccine campaign, and reduce vaccine refusal rates. Efforts to improve health communication in Pakistan are underway. UNICEF, for instance, has partnered with imams to facilitate immunisation in thousands of schools and madrassas across the country, and launched media campaigns to highlight the risks of polio and the importance of vaccination [51]. However, Pakistan’s government has also taken the controversial step of arresting parents who refuse to vaccinate their children; however, the utility of this approach in combating the negative narratives around vaccination are seriously open to question [30, 52, 53].

Pakistan’s Civil Society Organisations (CSOs), many of which provide community health services in regions without a public health infrastructure, can also be recruited to support campaign efforts. Many of these CSOs have already built strong partnerships with the communities they serve, are privy to local concerns and have the logistics to facilitate the immunisation campaign and associated health initiatives [19].

### Prioritising vaccination in polio-endemic regions

Pakistan’s NEAP includes an SIA prioritisation framework which classifies districts across the country into four tiers based on the incidence of poliomyelitis. It identifies 12 districts including Karachi, Quetta, parts of FATA and KP as polio-endemic ‘Reservoir Districts’ and 30 ‘High Risk Districts’ mainly in southern FATA and KP, which are scheduled to receive intensified SIA campaigns. In addition, 11 ‘Outbreak Districts’ have been added to the Sub-National Immunisation Day calendar. Low-risk districts covering the rest of Pakistan will follow the National Immunisation Day calendar. Mobile populations including IDPs are to be vaccinated at Permanent Transit Points (PTPs) in districts bordering KP and FATA, and at the border with Afghanistan; a crucial step in stemming the spread of polio to other parts of the country [43]. By coordinating these efforts to improve the polio eradication campaign with military offensives in FATA and KP, it is hoped that a significant reduction in poliomyelitis cases can be achieved by the end of 2016.

### Global health, diplomacy and foreign policy

One of the major lessons from the conflict and health interface in Pakistan is how security and health agendas can both fail and support each other. The clandestine use of a fake vaccination campaign in the search for Osama bin Laden significantly damaged the credibility of the GPEI and other vaccination programmes in Pakistan [33]. This is not an isolated incident, and several countries are alleged to have used aid workers for intelligence purposes [54]. The covert use of health initiatives to advance ulterior security or foreign policy motives inevitably undermines the safety of healthcare personnel, increases suspicion and hostility to such programmes among recipient countries, jeopardising their legitimate long-term global health goals [33, 40, 55].

Global health initiatives are becoming increasingly intertwined with the diplomatic, foreign policy and security interests of both donor and recipient countries and there are calls for greater cooperation between global health professionals and foreign policymakers to address this paradigm shift [40, 55]. Kaufmann and Feldbaum [49] detail how an integrated, nuanced approach towards the religious and political issues underlying the Northern Nigerian polio immunisation boycott helped resolve the crisis. Influential Islamic countries such as Saudi Arabia, which has extensive experience in implementing public health measures using formal religious authority, and international bodies such as the OIC and the International Fatwa Body can bolster Pakistan’s efforts in combating the religious issues underlying vaccine hesitancy in Pakistan [56].

More widely, global health programmes cannot isolate themselves from local and international economic, security and political interests. The USA, UK and European Union have all incorporated aid and ‘smart global health’ as an instrument of foreign policy, so too have emerging powers such as China, Brazil and Cuba [57]. However, the design and delivery of many of these programmes have failed to mitigate how they impact traditional social and religious norms, antagonising the recipient population and potentially undermining any health benefits. In the Pakistani context, formal liaisons do exist between the GPEI and the Pakistani government. The Pakistani military for instance has worked with vaccination teams to immunise IDPs fleeing conflict regions in FATA, and the immunisation schedule has been intensified for the 2015–16 period to target vulnerable populations; a good example of how security and health interests can align [43].

An important lesson from Pakistan’s case is how the exclusive use of health outcomes failed to realise the collateral and indirect effects on local cultural and religious sensibilities [58]. The NEAP outlines efforts to address these concerns, including support from media organisations and community outreach programmes to improve vaccine education among recipients [43]. However, addressing the long-term health inequalities in Pakistan (as argued elsewhere in this article) will require an overhaul of the health bureaucracy as well as improve access to health in hard-to-reach areas. Global health initiatives have the potential to facilitate these changes by aligning their primary health goals with local health priorities; partnering with CSOs and other organisations aimed at more general social welfare. Indeed Kevany [57, 58] has strongly argued that the inclusion of broader diplomatic, foreign policy and economic parameters in the evaluation, design and delivery of global health programmes can help establish standards and protocols through which primary health goals as well as collateral non-health diplomatic and security goals can be realised in a more acceptable and transparent manner.

## Conclusions

**Table:** 

PANEL: SECURITY, FOREIGN POLICY AND HEALTH
• Global health initiatives and foreign policy have become increasingly interlinked. The USA, UK and European Union have all incorporated aid as an instrument of foreign policy, so too have emerging powers such as China, Brazil and Cuba. • The alignment of health and foreign policy objectives can produce good outcomes, as was demonstrated by the US role in reducing Egyptian child mortality rates, and the international response to the Ebola crisis. • Diplomatic approaches have also been effective in resolving complex social, political and religious concerns related to polio vaccination such as Northern Nigeria’s polio boycott. Similar approaches can be useful in combating anti-vaccination propaganda in Pakistan. • However, using health projects to fulfil narrow security interests can seriously threaten the credibility of global health initiatives, compromising the safety of global health workers and undermining their progress. • Increased collaboration between foreign policymakers, diplomats and global health professionals can establish appropriate standards and protocols to account for socioeconomic, political, foreign policy and primary health interests when designing global health projects and help mitigate negative health and security consequences.

Pakistan’s polio eradication campaign is facing a range of challenges due to a poor health infrastructure, managerial and operational deficits and serious inequities in immunisation coverage across the country. Conflict and insecurity have been particularly damaging to polio eradication efforts in recent years, especially in FATA and KP, the main reservoirs of wild poliovirus in the country. Operation Zarb-e-Azb, a military offensive launched in 2014 against militants in FATA, has re-established access to vaccination teams and enabled more intensive SIAs, translating to a significant reduction in reported cases of poliomyelitis in 2015. Pakistan’s polio eradication effort can capitalize on these gains by addressing the gross inequities in its strategy and infrastructure, help shift perceptions against vaccination through more concerted community engagement and education initiatives, and address vaccine hesitancy using tools such as mass-media campaigns. Strengthening collaborations with influential religious leaders and organisations can also help mitigate the religious and political dimensions of vaccine hesitancy in Pakistan. Addressing these social and systemic deficits is critical to eradicating polio from Pakistan and achieving the GPEI’s objectives.

Pakistan’s polio eradication campaign provides important lessons for the delivery of future global health initiatives; the role of traditional social and religious norms and their wider diplomatic, security, economic and social repercussions in an era of increasing globalisation should not be underestimated in achieving global health outcomes and fostering broader socioeconomic development.

## Abbreviations

AFP: Acute Flaccid Paralysis
BHU: Basic Health Unit
CDC: Centers for Disease Control and Prevention
CIA: Central Intelligence Agency
CSO: Civil Society Organisation
cVDPV: Circulating Vaccine-derived Poliovirus
DPT: Diphtheria, Pertussis and Tetanus
EPI: Expanded Programme on Immunisations
FATA: Federally Administered Tribal Areas
GAVI: Global Alliance for Vaccinations and Immunisations
GPEI: Global Polio Eradication Initiative
IPV: Inactivated Polio Vaccine
KP: Khyber Pakhtunkhwa
NEAP: National Emergency Action Plan for Polio Eradication
NITAG: National Immunisation Technical Advisory Group
OIC: Organisation of the Islamic Conference
OPV: Oral Polio Vaccine
PTP: Permanent Transit Point
SIA: Supplementary Immunisation Activity
UNICEF: United Nations Children’s Emergency Fund
VAPP: Vaccine-associated Paralytic Poliomyelitis
WHA: World Health Assembly
WHO: World Health Organization
